# Artificial Intelligence-Assisted Colonoscopy for Detection of Colon Polyps: a Prospective, Randomized Cohort Study

**DOI:** 10.1007/s11605-020-04802-4

**Published:** 2020-09-23

**Authors:** Yuchen Luo, Yi Zhang, Ming Liu, Yihong Lai, Panpan Liu, Zhen Wang, Tongyin Xing, Ying Huang, Yue Li, Aiming Li, Yadong Wang, Xiaobei Luo, Side Liu, Zelong Han

**Affiliations:** Department of Gastroenterology, Nanfang Hospital, Southern Medical University, Guangzhou, 510515 China

**Keywords:** Colonoscopy, Artificial intelligence, Computer-aided diagnose

## Abstract

**Background and aims:**

Improving the rate of polyp detection is an important measure to prevent colorectal cancer (CRC). Real-time automatic polyp detection systems, through deep learning methods, can learn and perform specific endoscopic tasks previously performed by endoscopists. The purpose of this study was to explore whether a high-performance, real-time automatic polyp detection system could improve the polyp detection rate (PDR) in the actual clinical environment.

**Methods:**

The selected patients underwent same-day, back-to-back colonoscopies in a random order, with either traditional colonoscopy or artificial intelligence (AI)-assisted colonoscopy performed first by different experienced endoscopists (> 3000 colonoscopies). The primary outcome was the PDR. It was registered with clinicaltrials.gov. (NCT047126265).

**Results:**

In this study, we randomized 150 patients. The AI system significantly increased the PDR (34.0% vs 38.7%, *p* < 0.001). In addition, AI-assisted colonoscopy increased the detection of polyps smaller than 6 mm (69 vs 91, *p* < 0.001), but no difference was found with regard to larger lesions.

**Conclusions:**

A real-time automatic polyp detection system can increase the PDR, primarily for diminutive polyps. However, a larger sample size is still needed in the follow-up study to further verify this conclusion.

**Trial Registration:**

clinicaltrials.gov Identifier: NCT047126265

## Introduction

Colorectal cancer (CRC) is the third most commonly diagnosed malignancy and one of the leading causes of cancer-related death.^1^ Colonoscopy is the primary method for detecting and removing polyps. The detection and resection of tumor lesions by colonoscopy have been shown to be effective for the prevention of CRC.^2^ There is evidence that for every 1.0% increase in the rate of adenoma detection, the risk of CRC decreases by 3.0%.^3^ However, colonoscopy is not perfect, and occasionally, interval cancer is detected in patients with a recent normal colonoscopy.^4^ Due to the characteristics of polyps and operators, polyps are prone to missed diagnosis, and the missed diagnosis rate can be as high as 27%.^5^,^6^ Two factors are considered to affect the missed diagnosis rate: blind spots and human error. The first factor can be addressed by using a wide-angle range or wide-angle remote attachment, but human error is difficult to overcome. A physician’s workload may influence his/her level of performance.^7^ Even when the colonic mucosa is fully exposed, polyps may be missed because they are small or flat or because the color difference between the polyp and the normal mucosa is very small. In a 2006 systematic review and meta-analysis, six tandem colonoscopies showed a cumulative missed diagnosis rate of 22% for all polyps. This condition varied according to the size of the adenoma. The total missed diagnosis rate for adenomas ≥ 10 mm was 2.1%, that of adenomas 5–10 mm was 13%, and that of adenomas 1–5 mm was 26%.^8^ These findings emphasize that small polyps are prone to missed diagnosis regardless of experience. Furthermore, some studies have shown that the rate of polyp detection can be improved with the help of a second observer.^9^,^10^ Ideally, a real-time automatic polyp detection system with a performance similar to that of an expert endoscopist can be applied to help endoscopists detect polyp lesions; additionally, artificial intelligence (AI) has attracted attention in this area.^11^ To further reduce the missed diagnosis of colonic polyps, further technical progress is needed to optimize the detection and endoscopic evaluation of colonic polyps. Computer-aided diagnosis (CAD) can take advantage of progress in the field of AI, especially deep learning technology, and play an auxiliary role in colonoscopy, thus providing a promising solution for human performance changes. Deep learning patterns depend on artificial neural networks, which are inspired by the concept of the network of neurons and synapses in the human brain. For image analysis, the best results so far have been obtained using a model based on convolutional neural networks (CNNs), which consist of several simple computing nodes and complex connections to simulate the human visual cortex.^12^ CAD is a system that encompasses the ability of a computer to learn and perform specific tasks. Several automatic polyp detection systems have been developed in the past decade^13^,^14^; however, there is little evidence demonstrating the ability of this technique to locate and track polyps in real time during colonoscopy in clinical practice.

The purpose of this study was to explore whether AI-assisted colonoscopy could improve the polyp detection rate (PDR) in the actual clinical environment.

## Methods

### Patients

This study was conducted at the Endoscopy Center of Nanfang Hospital, China. Consecutive patients who underwent colonoscopy between April 2019 and September 2019 were eligible for enrollment. The specific inclusion criteria were as follows: (I) Chinese patients between the ages of 18 and 70 years; (II) voluntary signature of an informed consent form; and (III) no colonoscopy or anesthesia-related contraindications. The exclusion criteria were as follows: (I) history of inflammatory bowel disease (IBD); (II) history of colorectal surgery; (III) previously failed colonoscopy; (IV) polyposis syndrome; and (V) highly suspected CRC. In the course of the study, the following circumstances allowed patients to withdraw: (I) inadequate intestinal preparation; (II) withdrawal of informed consent by the patient; and (III) obvious adverse events that interrupted or affected the continuation of the examination. Basic demographic characteristics, including age, sex, and the Boston Bowel Preparation Scale (BBPS) score, were recorded.

### Study Design

**Fig. 1:**
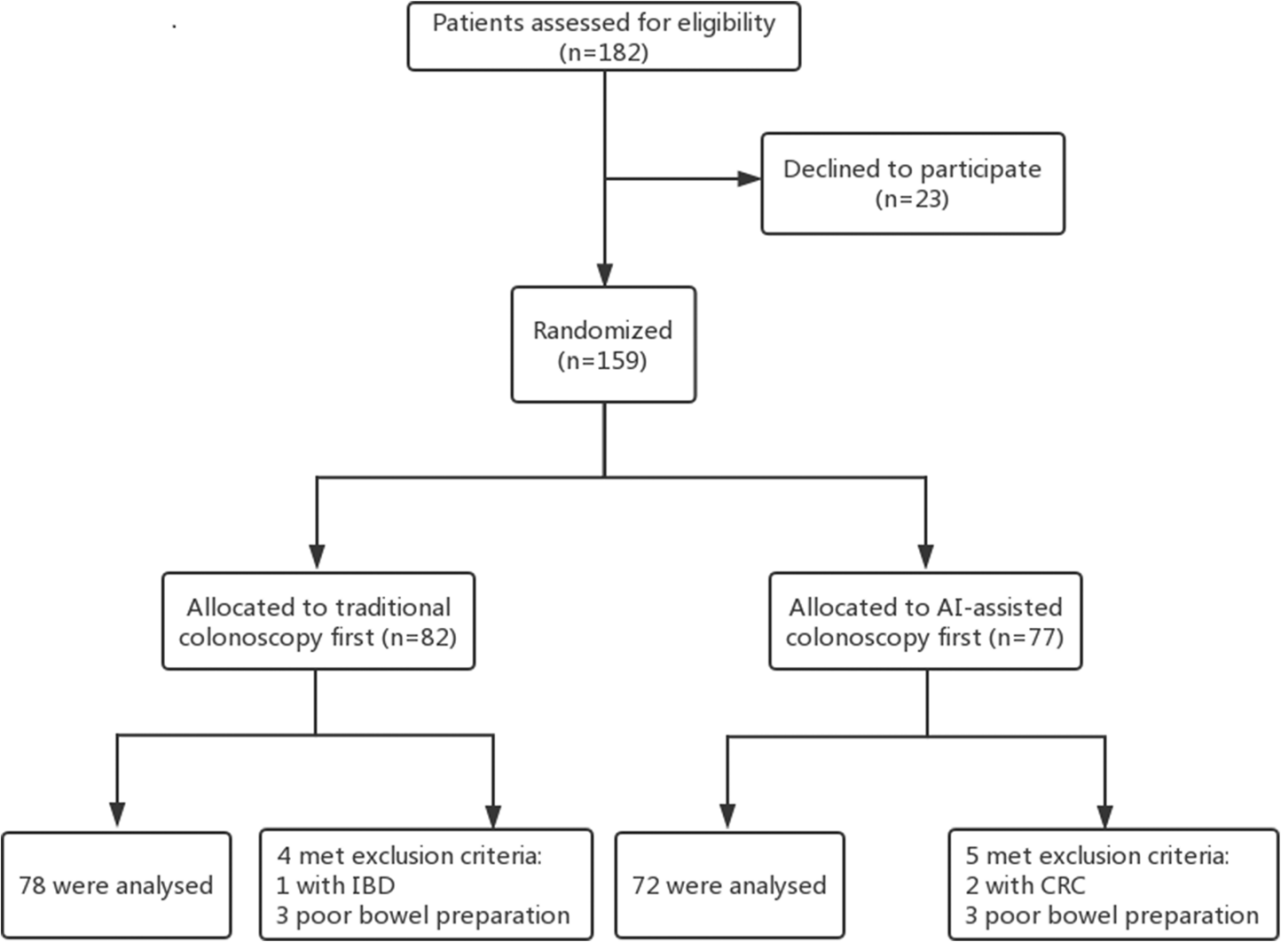
Flow diagram of enrollment. A total of 150 patients were analyzed, of whom 72 underwent AI-assisted colonoscopy first and 78 underwent traditional colonoscopy first

**Fig. 2:**
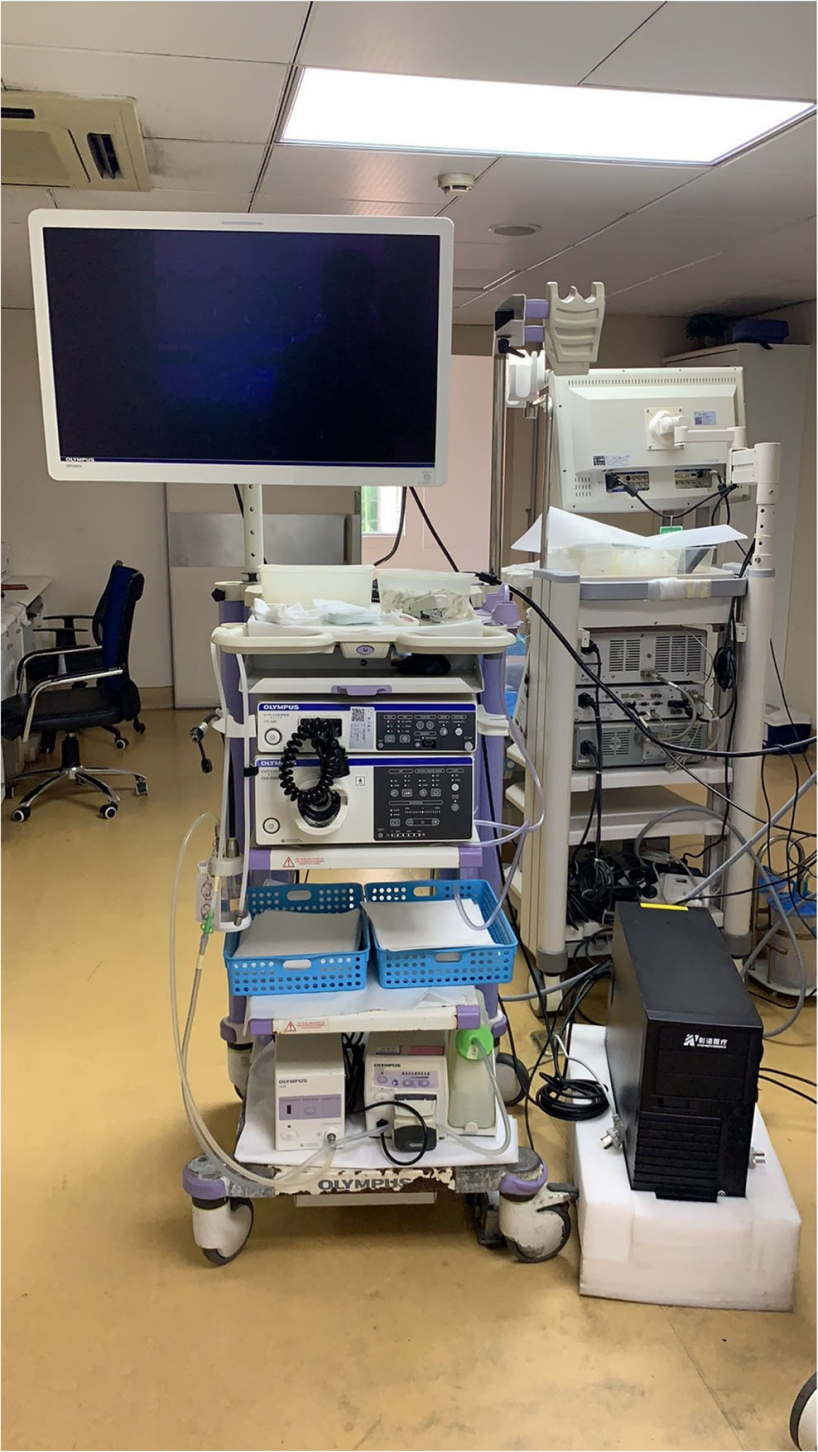
Host device of the real-time polyp detection system

**Fig. 3:**
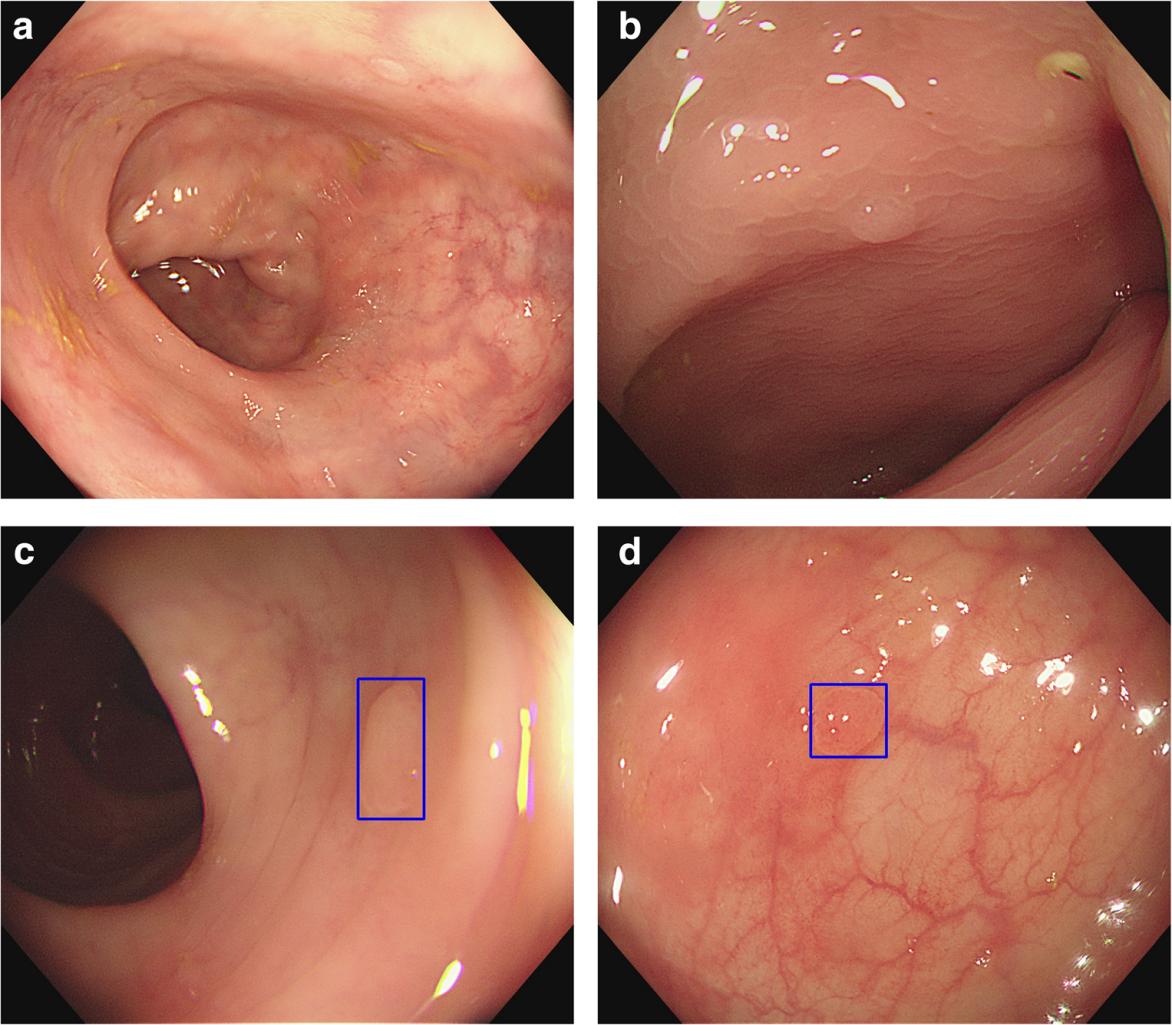
**a** and **b** Identification of polyps by traditional colonoscopy. **c** and **d** Blue box that appears when a polyp is identified by AI-assisted colonoscopy

This was a prospective cohort trial. The conventional colonoscopy group was the control group, and the AI-assisted colonoscopy group was the research group. All included patients were required to sign an informed consent form before the screening. Routine bowel preparation consisted of 4 L of polyethylene glycol given in split doses. Colonoscopies were performed with high-definition colonoscopes (Olympus 290, CF-HQ290ZI) and high-definition monitors. The real-time automatic polyp detection system adopted was obtained from Xiamen Innovision Co., Ltd. (Fig. 2). All procedures were carried out by two experienced endoscopists, each having performed more than 3000 standard colonoscopies. The patients were assigned, by digital random number generators, to undergo back-to-back tandem colonoscopies with either conventional colonoscopy or AI-assisted colonoscopy first, followed immediately by the other procedure performed by the other endoscopist. Two endoscopists performed each colonoscopy independently, and the patient’s final colonoscopy report was combined with the endoscopy results of the two doctors. Patients in the control group underwent routine colonoscopy (Figs. 1 and 2). In the research group, the real-time automatic polyp detection system was used for endoscopic assistance. The system, turned on during withdrawal only, was connected to the endoscope generator and synchronously captured the video stream. The system displayed the position of the detected polyp with a hollow blue tracking box on the adjacent monitor (Fig. 3). Polyps detected during the first procedure were left in situ to be removed at the end of the second procedure. The research was registered with clinicaltrials.gov (NCT047126265).

The primary outcome was the PDR. The secondary outcomes were the number of polyps detected, the number of diminutive polyps (diameter < 6 mm), the number of polyps of each Paris type detected, and the number of false-positive results.

### Statistical Analysis

We prospectively designed this study to allow 90% power or more to detect a 20% difference (45% vs 25%) between the colonoscopy procedures with a two-group *χ*2 test and a two-sided *α* level of 0.05. A total of 118 samples were required based on the paired *χ*2 difference test. A sample size of 118 participants was needed, and the overall participant enrollment goal was set at 157 to allow for potential exclusions or dropouts.

Measurement data are described as the mean and standard deviation. Count data are described as the number and percentage of patients. Comparisons of baseline and demographic characteristics between the research group and the control group were performed using the paired *χ*2 test (McNemar’s test) or Fisher’s exact test for categorical variables and using the paired *t* test for continuous variables. A *p* value less than or equal to 0.05 was considered to indicate a statistically significant difference between the experimental and control groups. The observational indexes of the two groups were statistically analyzed by SPSS 23.0 statistical software.

### The AI-Assisted System

**Fig. 4:**
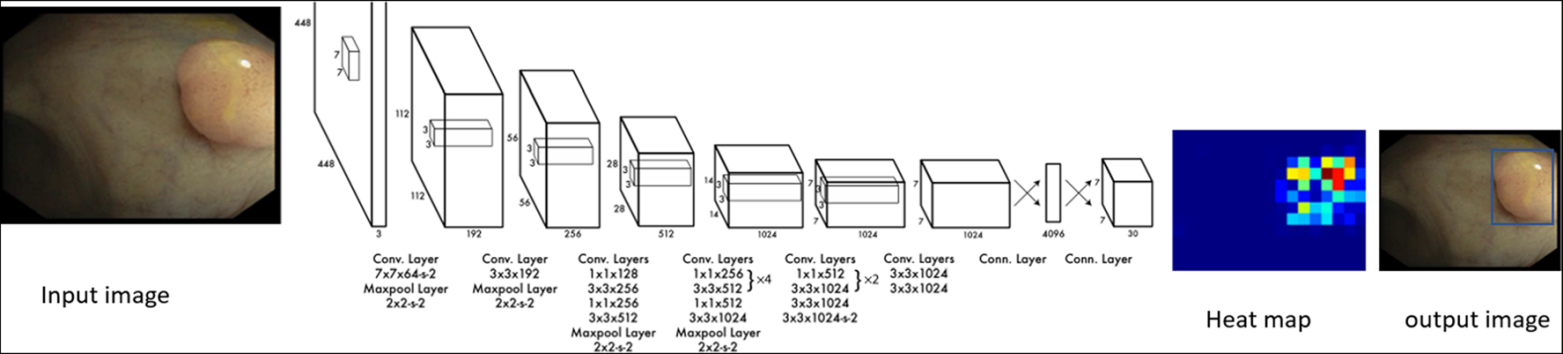
Artificial intelligence (AI) system. The detection algorithm is a deep convolutional neural network (CNN) based on the YOLO network architecture

The AI-assisted system (Xiamen Innovision Co., Ltd.) was developed by employing a CNN algorithm, specifically, a YOLO network architecture for object detection (Fig. 4). The model was trained on 112,199 colonoscopy images (including 64,134 images with 69,716 polyps and 48,065 images without polyps). All the images were recorded in the white light model and labeled by colonoscopists with more than 5 years of experience.

**Fig. 5:**
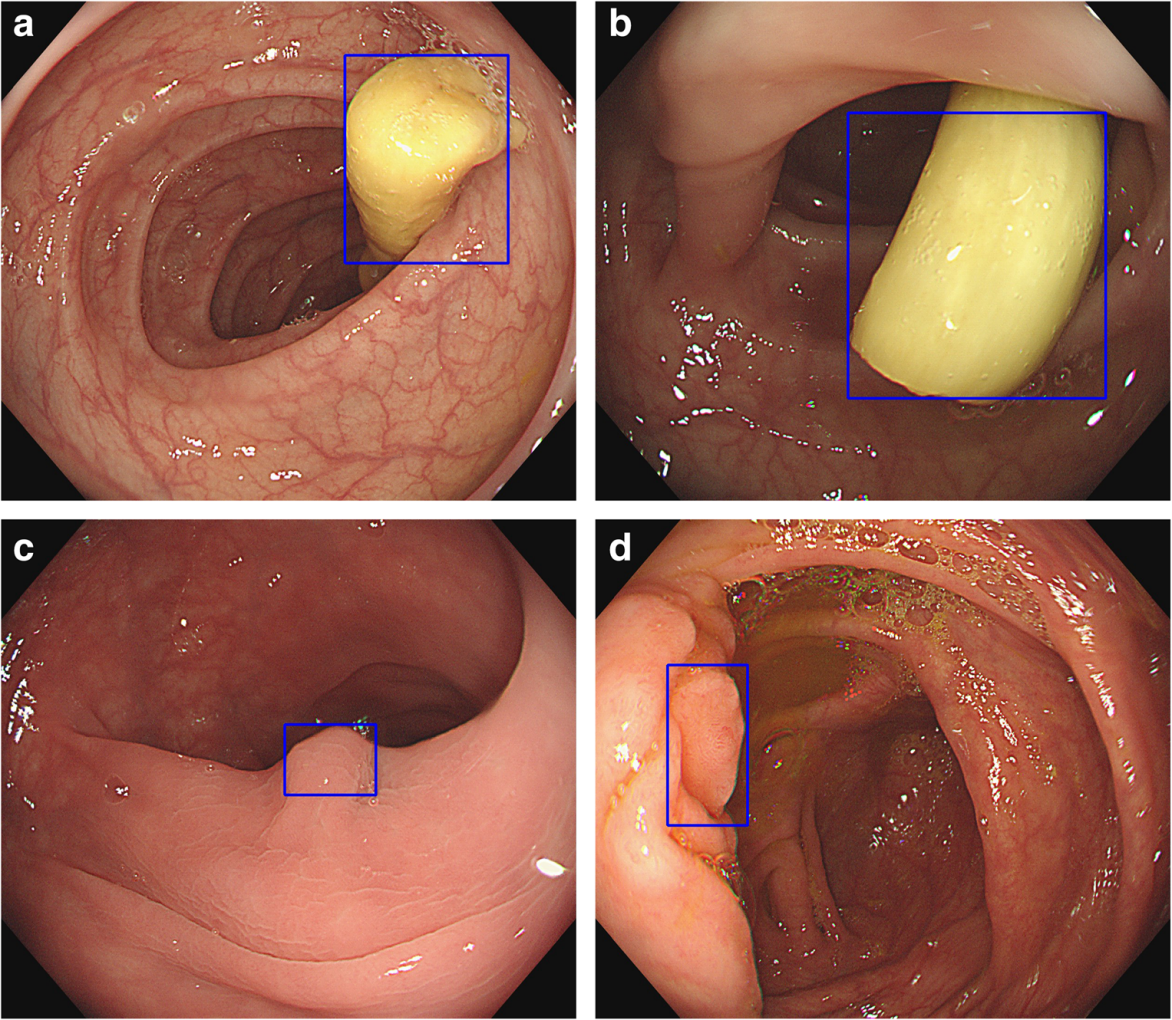
**a** and **b** Feces mistakenly identified by AI. **c** Mucosal fold mistakenly identified by AI. **d** Ileocecal lobe misrecognized by AI

To enable the trained model for real-time detection, we captured each frame of the colonoscopy video during a colonoscopy and sequentially sent the frames to a PC. The model was then called to analyze the colonoscopy frame by frame in the PC and display the results on the adjacent monitor. The hardware configuration of the PC contains an NVIDIA GeForce GTX 2080 Ti graphics card, an i7-6600 CPU, and an 8-GB memory. The whole system, including the software and hardware, can process at least 50 frames per second with a latency of 33.20 ± 10.13 ms in real-time video analysis (Fig. 5).

## Results

### Clinical Characteristics

A total of 150 people (76 males and 74 females) met the inclusion criteria and were therefore considered for enrollment in the study. Baseline characteristics are presented in Table 1. The average age of the patients was 41.3 years, with a maximum age of 68 years and a minimum age of 18 years. Each patient was evaluated for bowel preparation at the first withdrawal. According to the BBPS score, 84% of the patients scored greater than or equal to 6, and 16% of the patients scored less than 6. In these patients, a good examination could still be performed after water flushing and full suction, so they were not excluded. The average withdrawal time was 370.15 ± 31.44 s in the traditional enteroscopy group and 373.17 ± 33.37 s in the AI-assisted enteroscopy group (*p* = 0.102). Overall, there were no statistically significant differences between the two groups in terms of demographic data.

**Table 1:** Baseline characteristics of patients

Patient characteristic	Traditional colonoscopy (*n* = 150)	AI-assisted colonoscopy (*n* = 150)	*p* value
Withdrawal time, seconds, mean **±** SD	370.15 ± 31.44	373.17 ± 33.37	0.102
Age, years, mean ± SD (range)	41.3 ± 10.4 (18-68)	41.3 ± 10.4 (18-68)	1.000
Sex			
Male, *n* (%)	76 (51)	76 (51)	1.000
Female, *n* (%)	74 (49)	74 (49)	1.000
Bowel preparation score			
< 6, *n* (%)	24 (16)	24 (16)	1.000
≥ 6, *n* (%)	126 (84)	126 (84)	1.000

### Polyp Characteristics

There was no significant difference in the location, size, or classification of polyps between the two groups (Table 2). The number of polyps detected in the control group and the research group was 80 and 105, respectively (*p* = 0.020). The PDRs of the control group and the research group were 34.0% and 38.7%, respectively (*p* < 0.001) (Table 3). Significant differences were observed in the number of polyps and the PDR between the two groups. Furthermore, we classified statistics and compared them according to the size and the Paris classification of the polyps. According to the size of the polyps, 69 and 91 diminutive polyps were found in the control group and in the research group, respectively. The positive rates of diminutive polyp detection in the control group and the research group were 30.0% and 34.7%, respectively (*p* < 0.001). In contrast, there was no significant difference in the number of polyps larger than 6 mm (11 vs 14, *p* = 0.319). By comparing polyp morphology according to the Paris classification system, we observed a significant difference in the number of 0-IIa polyps between the two groups (61 vs 87, *p* = 0.010). AI-assisted colonoscopy is more sensitive to the detection of Paris type 0-IIa polyps.

**Table 2:** Polyp characteristics

Variable	Traditional colonoscopy (*n* = 80)	AI-assisted colonoscopy (*n* = 105)	*p* value
Location of polyp			0.764
Left colon, *n* (%)	44 (55.00)	57 (54.29)	0.923
Transverse colon, *n* (%)	22 (27.50)	33 (31.43)	0.562
Right colon, *n* (%)	14 (17.50)	15 (14.28)	0.551
Polyp size			0.935
<6 mm, *n* (%)	69 (86.25)	91 (86.67)	
≥6 mm, *n* (%)	11 (13.75)	14 (13.33)	
Polyp type^a^			0.352
0-IIa, *n* (%)	61 (76.25)	87 (82.86)	0.266
0-Is, *n* (%)	8 (10.00)	5 (4.76)	0.167
0-Ip, *n* (%)	11 (13.75)	13 (12.38)	0.784

^a^Paris classification

**Table 3:** Polyp detection

Variable	Traditional colonoscopy (*n* = 150)	AI-assisted colonoscopy (*n* = 150)	*p* value
Patients with at least one polyp, *n* (PDR)	51 (34.0%)	58 (38.7%)	< 0.001
Patients with at least one diminutive polyp, *n* (%)	45 (30.0%)	52 (34.7%)	< 0.001
Patients with at least one Paris type 0-IIa polyp, *n* (%)	39 (26.0%)	48 (32.0%)	< 0.001
Polyps detected, *n*	80	105	0.020
Polyps detected, by dimension			
< 6 mm, *n*	69	91	< 0.001
≥ 6 mm, *n*	11	14	0.319
Polyps detected, by type^a^			
0-IIa, *n*	61	87	0.010
0-Is, *n*	8	5	0.181
0-Ip, *n*	11	13	0.319

^a^Paris classification

### False Positives with the Automatic Polyp Detection System

There was a total of 52 false positives in the AI-assisted colonoscopy group, averaging 0.35 false positives per colonoscopy (Table 4), mostly due to feces, mucosal folds, and so on.

**Table 4:** Statistical results of false positives

	AI-assisted colonoscopy, *n* (%)
False positive	52 (100.00)
Submucosal tumor	4 (7.69)
Cyst	1 (1.92)
Feces	29 (55.77)
Ulcer	4 (7.69)
Mucosal fold	10 (19.23)
Other (bubble, circular blood vessel, and so on)	4 (7.69)

## Discussion

This was a prospective cohort study conducted to investigate the advantages of AI-assisted colonoscopy. Compared with the study by Wang et al.,^15^ our study ruled out the interference caused by intestinal differences between patients. Our study compared the number and positive detection rate of colorectal polyps between traditional colonoscopy and AI-assisted colonoscopy by observing withdrawal by different doctors in the same patient. Furthermore, because the order of the two procedures was completely random and the two operators were experienced endoscopists with the same level of seniority, the influence of the order of the procedure and the experience level of the operators on the results were minimized.

In this study, we found that AI-assisted colonoscopy could significantly increase the number of polyps detected compared with traditional colonoscopy, and the PDR of AI-assisted colonoscopy was also significantly higher than that of traditional colonoscopy. Moreover, we found that AI-assisted colonoscopy could significantly improve the detection of diminutive polyps (diameter < 6 mm). This may be because small polyps in the field of view are more likely to be missed by endoscopists, while larger polyps are more likely to be detected. Although smaller polyps have a lower risk of malignancy than larger polyps, an increase in the overall PDR may ultimately help reduce the risk of interval CRC. Further research can also focus on the role of CAD in decreasing the risk of interval CRC.^3^ However, the improved detection of diminutive polyps may lead to additional unnecessary polypectomies and add to the workload. In our study, there was no significant difference in the average withdrawal time between the two groups. It shows that the detection of more diminutive polyps by AI-assisted colonoscopy will not significantly increase the withdrawal time but also shows the real-time performance of CAD. Another study demonstrated that real-time CAD methods can achieve the performance level required for a diagnosis-and-leave strategy for diminutive, non-neoplastic rectosigmoid polyps,^16^ which could potentially reduce costs.

At the same time, we also classified and statistically analyzed the polyps according to the Paris classification system. Compared with traditional colonoscopy, AI-assisted colonoscopy showed a higher detection rate for 0-IIa polyps. This could be because most of the polyps were type 0-IIa and because the sample size was not large enough.

We found that the real-time polyp detection system yielded a total of 52 false positives, most of which were due to feces and mucosal folds. There were approximately 0.35 instances per colonoscopy. This may suggest that the AI system is highly sensitive. Moreover, the recognition of color, shape, and property by AI still needs to be improved. The AI-assisted colonoscopy group did not demonstrate a longer withdrawal time. These false alarm lesions could be eliminated by the identification of endoscopists.

Some limitations to this study need to be mentioned. First, this was a single-center study with a small sample size. Second, AI has different effects on improving the PDR among different doctors. Third, the trial did not compare the adenoma detection rate (ADR) between the two groups. In the future, we will conduct a trial to improve the limitations listed above and to explore the effect of AI-assisted colonoscopy on the PDR using doctors with different seniorities and with different ADRs. Improvements based on the above aspects will improve the AI algorithm and its application in the field of medicine.

In conclusion, this study shows that an AI system based on deep learning and its real-time performance led to significant increases in colorectal PDR. The study is the early stages of AI for polyp detection that may be clinically relevant and is justification for further investigation.
